# Comparative Mitogenomics of Wonder Geckos (Sphaerodactylidae: *Teratoscincus* Strauch, 1863): Uncovering Evolutionary Insights into Protein-Coding Genes

**DOI:** 10.3390/genes16050531

**Published:** 2025-04-29

**Authors:** Dongqing Zheng, Rongrong Ma, Xianguang Guo, Jun Li

**Affiliations:** 1 Xinjiang Key Laboratory of Biological Resources and Genetic Engineering, College of Life Science and Technology, Xinjiang University, Urumqi 830017, China; zhengdq9091@sina.com (D.Z.); 17799278874@163.com (R.M.); 2 Chengdu Institute of Biology, Chinese Academy of Sciences, Chengdu 610213, China; guoxg@cib.ac.cn

**Keywords:** mitochondrial genome, *Teratoscincus*, Sphaerodactylidae, protein-coding gene, evolution, phylogeny

## Abstract

**Background:** Comparative studies of selection pressures on mitochondrial genomes and protein-coding genes (PCGs) are scarce in the genus *Teratoscincus* (Strauch, 1863), particularly within Sphaerodactylidae. Given their close evolutionary relationship, *Teratoscincus przewalskii* (Strauch, 1887) and *Teratoscincus roborowskii* (Bedriaga, 1906) serve as ideal models for the characterization of mitochondrial genome sand analysis of selective pressure in this genus. **Methods:** In this study, we employed Sanger sequencing to sequence the mitochondrial genome of *T. roborowskii* (Bedriaga, 1906), and utilized sliding window analysis, selection pressure analysis etc. to compared it with that of its close relative, *T. przewalskii* (Strauch, 1887). **Results:** The results contain the genome composition, *Ka*/*Ks* values, AT/GC-skew, etc. Selection pressure analysis of PCGs across *Teratoscincus* (Strauch, 1863) species (including those in GenBank) revealed that most genes evolve slowly, with the exception of *ATP8* and *ND6*, which exhibited faster evolutionary rates. Notably, the *ND6* of *T. roborowskii* (Bedriaga, 1906) demonstrated rapid non-synonymous substitution rates which may contribute to the survival and reproductive success of the species by favoring advantageous mutations. Phylogenetic analysis for the mitochondrial genomes of Sphaerodactylidae, Phyllodactylidae, and Gekkonidae confirmed the distinctiveness of Sphaerodactylidae and the two *Teratoscincus* (Strauch, 1863) species. **Conclusions:** This study has advanced the understanding of adaptive evolution in *Teratoscincus* (Strauch, 1863) mitochondrial genomes, expanded the mitochondrial database of Sphaerodactylidae, and provided insights into the phylogenetic relationships of the genus.

## 1. Introduction

The wonder gecko genus *Teratoscincus* (Strauch, 1863), belonging to the family Sphaerodactylidae, is native to the desert regions of southwestern and central Asia [1,2]. Initially classified within the Gekkonidae, subsequent molecular, morphological, and anatomical evidence has led to its reclassification within the Sphaerodactylidae [3,4]. The genus *Teratoscincus* comprises nine known species, three of which occur in China: *T. roborowskii* (Bedriaga, 1906), *T. przewalskii* (Strauch, 1887), and *T. scincus* (Schlegel, 1858) [1,2,5,6,7].

*T. roborowskii*, the Turpan wonder gecko, is endemic to China’s Turpan Basin and is nocturnal, preferring habitats with dead trees of thorny bushes [8,9,10]. Once misclassified as *T. przewalskii*, it has recently been established as a separate species [2]. In contrast, *T. przewalskii* inhabits three major desert ecosystems in northwestern China: Tarim Basin, Hami Depression, and Gobi deserts [1,2]. This nocturnal species feeds primarily on beetles and occupies arid sandy or gravelly environments [11,12].

Despite its ecological and geographic importance, research on *Teratoscincus* remains limited, with most studies focusing on physiology, behavior, and phylogeny rather than mitochondrial genomics [1,2,10,13]. These species have distinct ecological niches and distribution patterns; *T. przewalskii* occupies a wide altitudinal range across northwestern China, while *T. roborowskii* is restricted to the Turpan Basin. This characteristic makes them an ideal comparative model for exploring mitochondrial genome dynamics at the interspecific level, particularly in adaptation to divergent desert environments.

The mitochondrial genome (mitogenome), pivotal to energy metabolism through oxidative phosphorylation [14], serves as a key molecular marker for genetic studies due to its matrilineal inheritance, simple structure, high copy number, and rapid evolutionary rate [15]. Its applications span phylogenetic analysis, population genetics, species identification, and taxonomic classification [16,17,18]. Typically comprising twenty-two tRNAs, two rRNAs, thirteen PCGs, and a control region [19,20], mitochondrial genomes have been extensively studied across diverse animal taxa [15,18,21,22].

Despite the extensive investigation, research on mitochondrial genomes within the genus *Teratoscincus* and the family Sphaerodactylidae remains limited, with existing studies primarily focusing on single species characterization rather than comparative analyses [12,23,24]. Further comparative research on *Teratoscincus* mitochondrial genomes could provide valuable insights into their evolutionary adaptations to desert environments.

The present study has, therefore, sought to provide a new complete mitochondrial genome of *T. roborowskii* and to compare it with available *Teratoscincus* mitochondrial genomes in GenBank, focusing in particular on *T. przewalskii*. The analysis included a range of aspects, including genome composition, gene order, base composition, and codon usage. A non-synonymous mutation was identified in the *ND6* between the two species, and further analysis was performed on *Ka*/*Ks* values, AT/GC-skew, and sources of selection pressure within the genus. Complete mitochondrial genome sequences were subjected to phylogenetic reconstruction using both maximum likelihood (ML) and Bayesian inference (BI) methods. The results of this study offer novel insights into the dynamics of selection pressure in the mitochondrial genomes of closely related species and expand the genetic resources available for *Teratoscincus* species.

## 2. Materials and Methods

### 2.1. Sampling and DNA Extraction

A tail-end sample of *T. roborowskii* (voucher number ZY01507) was collected in Toksun, Xinjiang (42.863° N, 88.633° E), China, in 2018. The sample was used for genetic analysis and preserved in 95% ethanol at −20 °C. It is currently deposited in the Chengdu Institute of Biology, Chinese Academy of Sciences. Genomic DNA was then extracted from the muscle tissue using the EasyPure Genomic DNA Kit (TransGen Biotech Co., Beijing, China) according to the manufacturer’s instructions. The integrity of the DNA was then assessed via 1% agarose gel electrophoresis.

### 2.2. Primer Design, PCR Amplification, and Sequencing

In order to amplify the mitochondrial genome of *T. roborowskii*, a set of 12 primer pairs was designed on the basis of published sequences from related species: *T. keyserlingii* (GenBank accession number AY753545) [1], *T. roborowskii* (MT107158) [25], and *T. przewalskii* (OL471044) [23].

PCR amplifications for target genes were performed with a volume of 25 μL, containing 12.5 μL of 2× Taq PCR Master Mix (Sangon Biotech, Shanghai, China), 0.5 μL of each specific primer pair (forward and reverse), 1 μL of template DNA (~50 ng), and 10.5 μL of sterilized ultrapure water. The PCR reactions were conducted as follows: an initial denaturation at 94 °C for 4 min, followed by 35 cycles of denaturation at 94 °C for 45 s, annealing at 48–54 °C for 35 s, extension at 72 °C for 90 s, and a final extension at 72 °C for 10 min. Assessment of the PCR products was undertaken via 1% agarose gel electrophoresis, after which the samples were sent to Sangon Biotech (Shanghai, China) for purification and sequencing. Sequencing was conducted using an ABI 3730 automated DNA sequencer (Applied Biosystems, Inc., Shanghai, China). Bidirectional sequencing was performed of all PCR products.

### 2.3. Assembly and Annotation

Raw sequences were proofread and assembled using BioEdit v7.2.5 [26]. The mitochondrial genome of *T. roborowskii* was automatically annotated using the MITOS WebServer (http://mitos.bioinf.uni-leipzig.de/index.py (22 December 2024)) [27]. Subsequently, using *T. roborowskii* (MT107158) as the reference genome, exact gene boundaries were further confirmed by comparing each gene with the annotated mitochondrial genomes from this species using the NCBI Blast online tool (https://blast.ncbi.nlm.nih.gov/Blast.cgi (22 December 2024)), followed by manual verification. The boundaries and length of control region (CR) were then determined based on the positions of *tRNA^phe^* and *tRNA^Pro^*.

### 2.4. Bioinformatics Analyses

The circular, complete mitochondrial genome of *T. roborowskii* was mapped using the MitoAnnotator online tool (https://mitofish.aori.u-tokyo.ac.jp/annotation/input/ (22 December 2024)) [28,29]. The nucleotide composition of the complete mitochondrial sequences, protein-coding genes (PCGs), RNAs, and CRs of the two species were calculated using MEGA v7.0 [30]. The AT-skew and GC-skew were calculated using the following formulae: AT-skew = ((A% − T%)/(A% + T%)); GC-skew = ((G% − C%)/(G% + C%)).

Synonymous substitutions (*Ks*) and non-synonymous substitutions (*Ka*) in PCGs were analyzed using BioEdit v7.0 [26] and KaKs Calculator v3.0 [31]. The effective number of codons (ENC) values for PCGs were calculated using the EMBOSS Explorer online tool (https://embossgui.sourceforge.net/demo/ (22 December 2024)). GC3s (GC content of the third position of synonymous codons) was calculated using CodonW v1.4.2 (https://codonw.sourceforge.net/ (22 December 2024)). Relative synonymous codon usage (RSCU) value was calculated in PhyloSuite v1.2.3 [32]. Pi analyses were performed using sliding window analysis in DNAsp v6.0 [33] to elucidate the variations and evolution in PCGs. All figures were created and enhanced using the ggplot2 package in R Studio v23.3.1 [34].

### 2.5. Phylogenetic Analysis

In order to establish the phylogenetic placements of *T. przewalskii* and *T. roborowskii*, 11 mitochondrial genome sequences of all available *Teratoscincus* and some related taxa were downloaded from GenBank (see Supplementary Table S1). Two Gekkonidae species were selected as outgroups: *Gekko gecko* (HM370130), *Gekko chinensis* (KP666135) [35].

Sequence alignment was performed using ClustalW implemented in BioEdit v7.0 [26], with manual adjustments. The partitioning schemes for maximum likelihood (ML) analysis was determined via the automated model screening functionality of IQ-TREE v2.2.2.6 [36]. For maximum likelihood (ML) analysis, the optimal model of the thirteen PCGs is GTR + F + R3. Based on the Akaike Information Criterion (AIC), the partitioning schemes of the thirteen PCGs for Bayesian inference (BI) analysis were established via the application of PartitionFinder v2.1.1 [37]. For Bayesian inference analysis, The mitochondrial DNA dataset was partitioned into 11 evolutionary units based on gene-specific characteristics, with optimal nucleotide substitution models determined for each partition: mtDNA *Cyt-b* and *ND1* (GTR + I + G + X), *ND2* (GTR + I + G + X), *COX1* (GTR + I + G + X), *COX2* (GTR + I + G + X), *ATP8* (GTR + I + G + X), *ND4L* and *ATP6* (GTR + I + G + X), *COX3* (GTR + I + G + X), *ND3* (HKY + I + G + X), *ND4* (GTR + I + G + X), *ND5* (GTR + I + G + X), and *ND6* (GTR + G + X). Bayesian inference was performed using MrBayes v3.2.7 [38] with two independent runs of two million generation each, sampling every 100 generations. Convergence of the MCMC runs was assessed using Tracer v1.7 [39], with diagnostic criteria set as follows: the average standard deviation of split frequencies < 0.01 and effective sample sizes (ESS) > 200 for all parameters. Thereafter posterior probabilities (PPs) were calculated from the combined samples of two independent runs, after the first 25% was discarded as burn-in. The tree topology was considered to have strong support of the PP was greater than 0.95. The ML tree was constructed using IQ-TREE v2.2.2.6 [36] with an ultra-fast bootstrap approximation of 4,000 replicates. Nodes with UFBoot support > 95% were considered to have strong support. Following this, the resulting phylogenetic trees were visualized and annotated using FigTree v1.4.4 (http://tree.bio.ed.ac.uk/software/figtree/ (22 December 2024)) and Microsoft PowerPoint 2010.

## 3. Results

### 3.1. Mitogenome Organization and Nucleotide Composition

**Figure 1:**
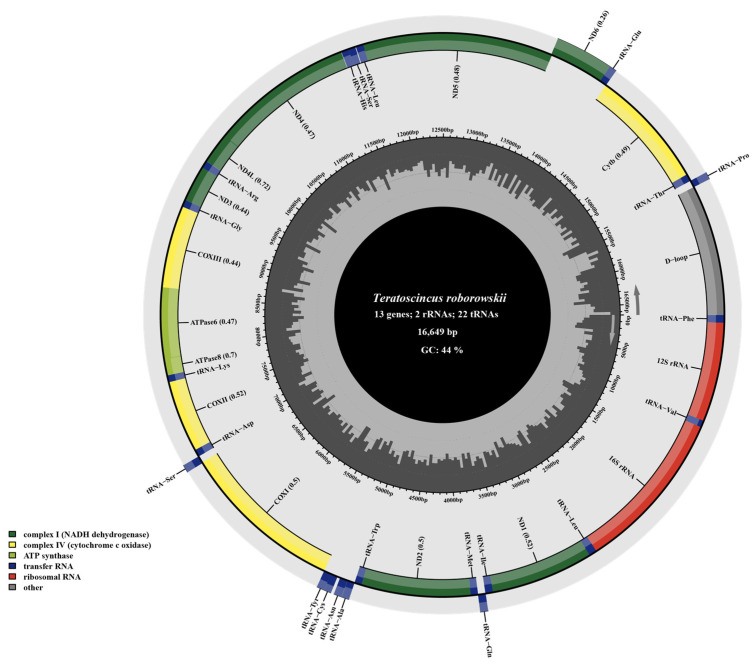
Mitochondrial genome map of *T. roborowskii* illustrates arrangement of genes encoded by both strands. Genes encoded by the H-strand are shown on the outside, while those encoded by the L-strand are indicated on the inside, with arrows showing their transcription direction. tRNAs, depicted in blue, are labelled according to three-letter amino acid codes. Innermost circle visualizes GC content across the mitochondrial genome, calculated every 5 bp. Darker lines represent regions with higher GC percentage.

The mitochondrial genome of *T. roborowskii* (16,649 bp) was sequenced and annotated, and compared with *T. przewalskii* (17,184 bp). The composition and the arrangement of mitochondrial genes in both species were found to be typical of most vertebrates (Figure 1, Supplementary Table S2). Each genome contained of thirteen PCGs, twenty-two tRNA genes, two rRNA genes (12S rRNA and 16S rRNA), and one non-coding region (the CR). Gene distribution analysis revealed strand asymmetry, with twenty-eight genes (including twelve PCGs, two rRNAs, and fourteen tRNAs) positioned on the heavy strand, while the remaining nine genes (*ND6* and eight tRNAs) resided on the light strand. The length of the origin of light-strand replication (OL) is 27 bp in *T. przewalskii* and *T. roborowskii* (Figure 1; Supplementary Table S2).

The mitochondrial genes in both species are tightly arranged, with some genes overlapping and only a few very short intergenic regions present (Supplementary Table S2). In *T*. *przewalskii,* overlapping gene pairs include: *tRNA^Ile^*-*tRNA^Gln^*, *tRNA^Gln^*-*tRNA^Met^*, *COXI*-*tRNA^Ser^*^(ACU)^, *ATP8*-*ATP6*, *ATP6*-*COXIII*, *ND4L*-*ND4*, and *ND5*-*ND6*. Of these, only three overlaps (*ATP8*-*ATP6*, *ATP6*-*COXIII*, *ND4L*-*ND4*) are on the same strand. The longest sequence overlap is a 10 bp sequence shared between *ATP8* and *ATP6*. In *T*. *roborowskii*, overlapping gene pairs are: *tRNA^Ile^*-*tRNA^Gln^*, *tRNA^Gln^*-*tRNA^Met^*, *COXI*-*tRNA^Ser^*^(CGA)^, *ATP8*-*ATP6*, *ATP6*-*COXIII*, *ND4L*-*ND4*, and *ND5*-*ND6*. Similar to *T. przewalskii*, only three pairs (*ATP8*-*ATP6*, *ATP6*-*COXIII*, *ND4L*-*ND4*) exhibit overlap on the same strand, with the most extensive overlap being 10 bp between *ATP8* and *ATP6*.

Nucleotide composition, AT skew, and GC skew were calculated for the total mitogenomes, PCGs, rRNAs, tRNAs, and CR of both species (Table 1). The mean AT content of the two complete mitochondrial genomes is almost similar: 55.8% in *T. przewalskii* and 56.2% in *T. roborowskii*. Both mitochondrial genomes showed a marginally positive AT-skew and a slightly negative GC-skew.

### 3.2. PCGs and Codon Usage

The mitochondrial genomes of all *Teratoscincus* species that have been sequenced thus far contained 13 PCGs (*ND2*, *COXI*, *COXII*, *ATP8*, *ATP6*, *COXIII*, *ND3*, *CYTB*, *ND5*, *ND4*, *ND4L*, *ND6*, and *ND1*). The range in length of these genes is from 165 bp (*ATP8*) to 1812 bp (*ND5*). The total length of the PCGs is 11,367 bp in (*T. przewalskii*) and 11,340 bp in *T. roborowskii*. Twelve of these PCGs (*ND2*, *COXI*, *COXII*, *ATP8*, *ATP6*, *COXIII*, *ND3*, *CYTB*, *ND5*, *ND4*, *ND4L*, and *ND1*) are encoded on the majority (H-) strand, while *ND6* is encoded on the minority (L-) strand. The start codons for the 13 PCGs in both *T. przewalskii* and *T. roborowskii* are primarily ATN (ATG, ATC, and ATA) and GTG. However, there are some differences in the usage of stop codons: the *COXI* in *T. przewalskii* uses AGG, while in *T. roborowskii* it uses AGA. The remaining genes typically use the standard TAN (TAG and TAA) stop codons. In addition, some genes have been observed to utilize incomplete termination codons, such as TA– and T––.

**Figure 2:**
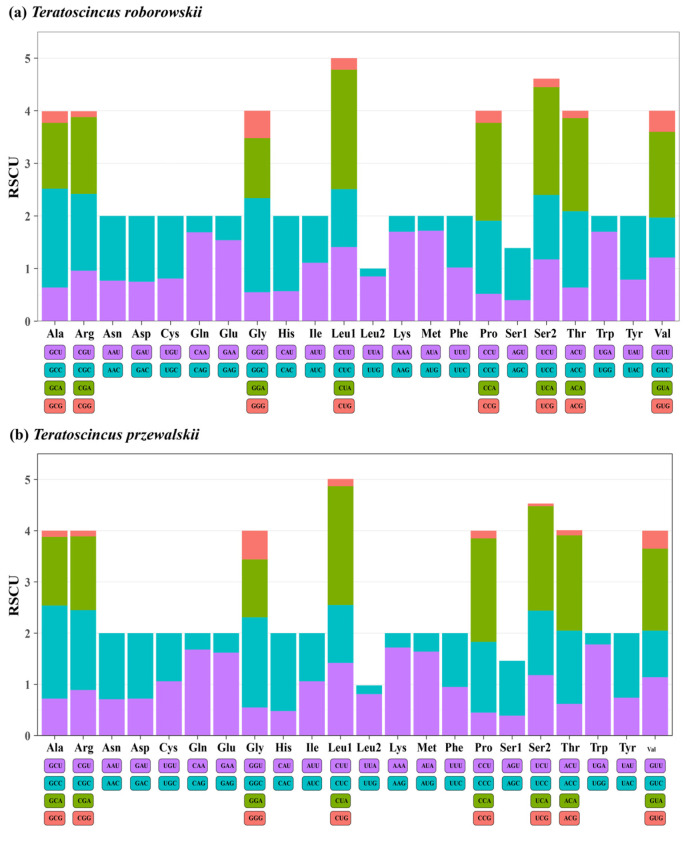
Base composition and relative synonymous codon usage (RSCU) values of *T. przewalskii* (**b**) *and T. roborowskii* (**a**).

The nucleotide composition of the three codon positions (including incomplete stop codons) of the 13 PCGs was found to be consistent between *T. przewalskii* and *T. roborowskii*. The third codon position exhibited the highest AT content, with 61.1% in *T. przewalskii* and 61.2% in *T. roborowskii*. For the first and third codons, the most prevalent nucleotide was A, at 30% in *T. przewalskii* and 29.9% in *T. roborowskii* for the first position, and 38.5% in *T. przewalskii* and 37.8% in *T. roborowskii* for the third position. In the second codon position, the most prevalent nucleotide was T, with 40.1% in *T. przewalskii* and 40.3% in *T. roborowskii*. Conversely, the third codon position exhibited a significantly lower frequency G, at 5.4% in *T. przewalskii* and 6% in *T. roborowskii*. AT-skew and GC-skew analyses revealed nucleotide usage patterns across codons, indicating a higher frequency of A at the first and third codon positions, a higher frequency of T at the second position, and a higher frequency of C at all three positions (Supplementary Table S3). These patterns align with the high A + T content and apparent AT-skew observed in PCGs (Figure 2).

The most frequently used codon was CUA (Leu), representing 6.82% of codons in *T. przewalskii* and 6.64% in *T. roborowskii*. Other frequently used codons included ACA (Thr) at 4.79% in *T. przewalskii* and 4.5% in *T. roborowskii*, and AUA (Met) at 4.28% in *T. przewalskii* and 4.5% in *T. roborowskii*, CUU (Leu) at 4.18% in *T. przewalskii* and 4.13% in *T. roborowskii*, and AUU (Ile) at 3.99% in *T. przewalskii* and 4.26% in *T. roborowskii.* In contrast, the least commonly used codons were identified as CGG (Arg), UCG (Ser2), CCG (Pro), and AAG (Lys) (Figure 2). These results indicate a preference for codons ending with A/T (U) in the mitochondrial PCGs of *Teratoscincus*.

**Figure 3:**
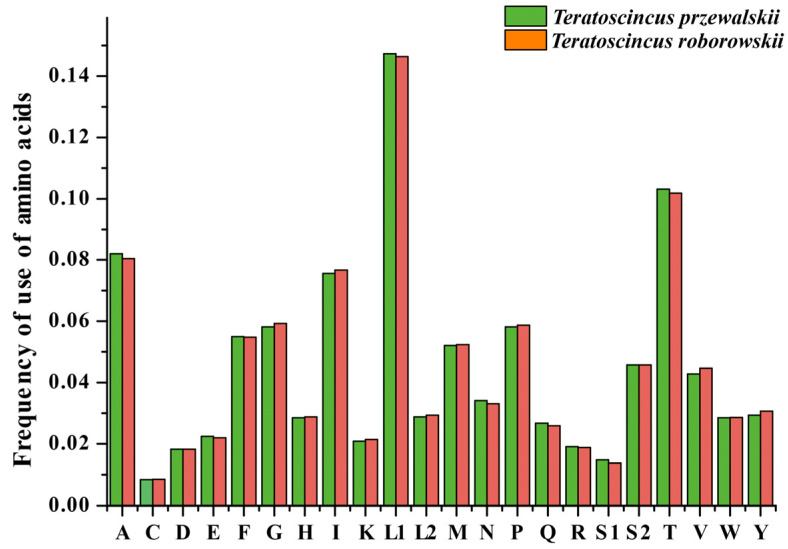
Frequency of use of amino acids in mitochondrial PCGs of *T. przewalskii* and *T. roborowskii*.

This tendency is further evidenced by the frequency of amino acid usage in these two species (see Figure 3), including Leucine (14.73% in *T. przewalskii* and 14.63% in *T. roborowskii*), Theonine (10.31% in *T. przewalskii* and 10.19% in *T. roborowskii*), Alanine (8.20% in *T. przewalskii* and 8.04% in *T. roborowskii*), and Isoleucine (7.56% in *T. przewalskii* and 7.67% in *T. roborowskii*). These findings highlight the evolutionary adaptation of *Teratoscincus* mitogenomes to elevated A/T content and AT-skew.

### 3.3. Comparative Analysis of Evolutionary Selection in Teratoscincus Species

**Figure 4:**
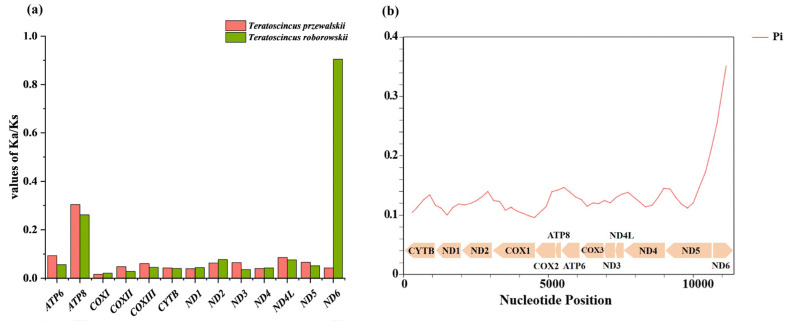
Variation in mitochondrial genes and evolutionary characteristics of *Teratoscincus*. (**a**) *Ka*/*Ks* values of mitochondrial gene sequences within *T. przewalskii* and *T. roborowskii*, revealing its evolutionary characteristics. (**b**) Sliding window analysis within *Teratoscincus*, revealing the nucleotide diversity (Pi). Arrow direction to the left indicates that the gene is located in the heavy chain, while arrow direction to the right indicates that the gene is located in the light chain.

The values of *Ka* (the number of non-synonymous substitutions per non-synonymous site), *Ks* (the number of synonymous substitutions per synonymous site), and the *Ka*/*Ks* ratio were calculated for each PCG in *T. przewalskii* and *T. roborowskii* (Figure 4). The *Ka*/*Ks* ratio for all 13 PCGs was found to below 1.0, indicating that these genes are evolving under purifying selection [40]. However, a notable deviation from this pattern was observed in the *Ka*/*Ks* ratio of *ND6*, which exhibited a significantly different pattern between the two species. In *T. roborowskii*, the rates of synonymous and non-synonymous substitutions in the *ND6* gene were almost identical. This finding indicates that the evolutionary rate of *ND6* was faster in *T. roborowskii* compared to other mitochondrial PCGs. Furthermore, the presence of non-synonymous mutations in this gene suggests that they are likely to be functional alterations that may contribute to the species’ adaptation to its environment (Figure 4).

**Figure 5:**
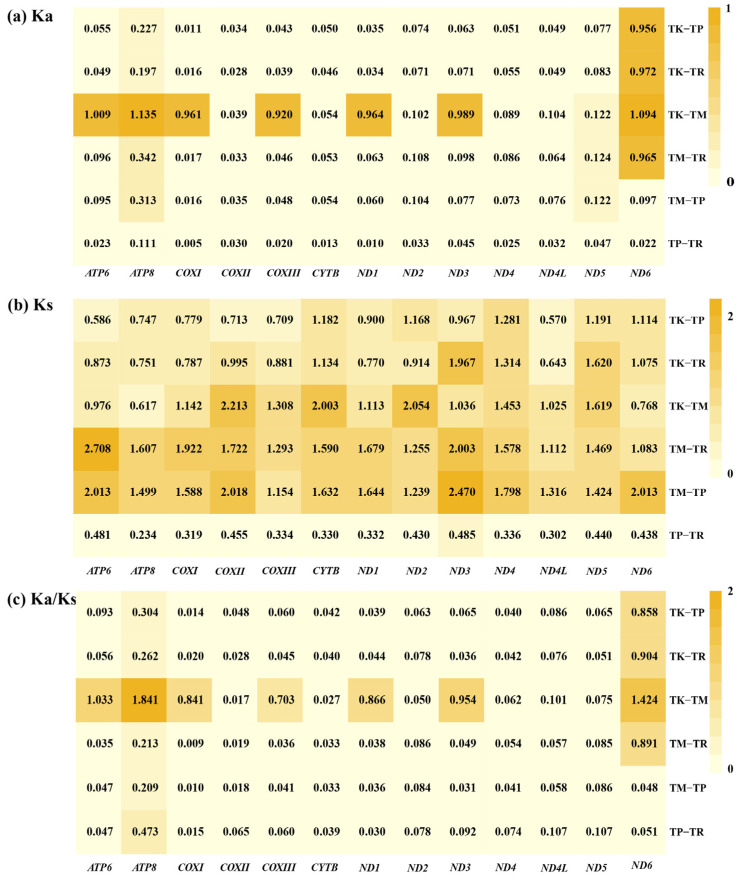
*Ka*/*Ks* values for each protein-coding gene (PCG) in pairwise mitochondrial genomes of four wonder geckoes. Abbreviations used are as follows: TP: *T. przewalskii*; TR: *T. roborowskii*; TK: *T. keyserlingii*; TM: *T. microlepis*. *Ka* values of PCGs of four wonder geckoes (**a**), *Ks* values of PCGs of four wonder geckoes (**b**), *Ka*/*Ks* values of PCGs of four wonder geckoes (**c**).

To further explore whether evolutionary selection for genetic preferences occurs in other species of *Teratoscincus*, a comparison was conducted of different species within the *Teratoscincus* (see Figure 4 and Figure 5). A sliding window analysis of nucleotide diversity (Pi) across the PCGs of the genus *Teratoscincus* revealed significant variation (Figure 4). The average Pi value of each gene ranged from 0.110 (*ND1*) to 0.309 (*ND6*). Specifically, *ND6* had the highest Pi value of 0.309, while *ATP8*, *ATP6*, and *ND5* exhibited the relatively higher Pi values of 0.144, 0.140 and 0.140, respectively. Conversely, *ND1*, *COXI*, and *COXII* had the lowest Pi values of 0.110, 0.111, and 0.114, respectively. These findings indicate that *ND6* and *ATP8* are highly variable genes, while *COXI* and *COXII* are more conserved within the genus.

A further investigation into the *Ka*/*Ks* values of four wonder geckoes (Figure 5) revealed significant variability. *T. keyserlingii* and *T. microlepis* exhibited relatively high variability in most PCGs, likely due to their distant affinities. *ATP8* and *ND6* consistently showed higher *Ka*/*Ks* ratios, indicating faster evolutionary rates, while *COXI* and *COXII* exhibited lower *Ka*/*Ks* ratios, suggesting slower evolutionary rates and stronger purifying selection.

Overall, the results suggest that *ATP8* and *ND6* are fast-evolving genes, potentially driven by adaptive selection or relaxed constraints. In contrast, *COXI* and *COXII* exhibited slower evolutionary rates, suggesting their function is subjected to strong purifying selection, likely in order to maintain essential physiological processes. This comparative analysis highlights the diverse evolutionary dynamics within the *Teratoscincus* mitochondrial genome.

### 3.4. AT/GC-Skew Analysis in Teratoscincus Species

**Figure 6:**
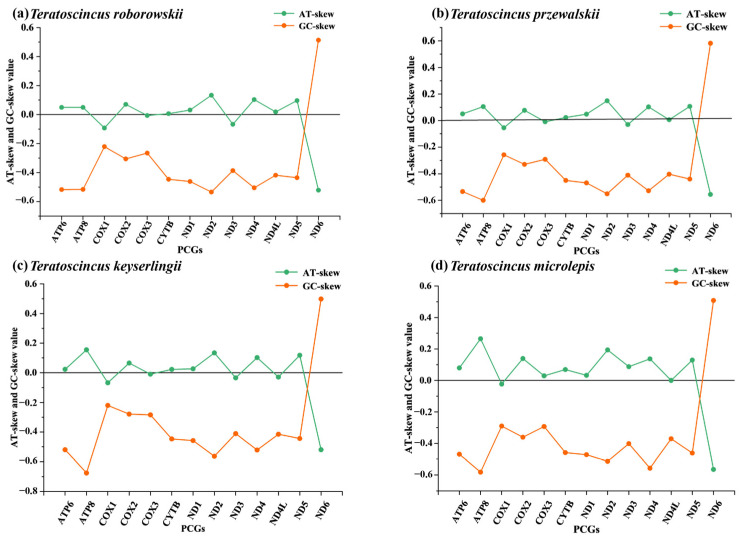
AT-skew and GC-skew values for four species. (**a**) *T. roborowskii*, (**b**) *T. przewalskii*, (**c**) *T. keyserlingii*, (**d**) *T. microlepis*.

Furthermore, the AT/GC-skew of four different species within the genus *Teratoscincus* was analyzed (Figure 6). The results showed that most of the AT-skew and GC-skew values of the four species in the genus *Teratoscincus* were negative. The content of T and C in the PCGs was greater than that of A and G. The difference in content between A and T was relatively small, while the difference in content between G and C was large, indicating an obvious GC bias and a slight AT bias. Among the four species of *Teratoscincus*, the variation between AT-skew and GC-skew was most evident in the *ND6* gene, followed by *ATP8* and *ND2*. This substantial fluctuation in AT/GC-skew in *ND6*, *ATP8*, and *ND2* is presumably associated with the selective and mutational pressures acting on these genes.

### 3.5. Driver of Codon Usage Bias in Teratoscincus Species

**Figure 7:**
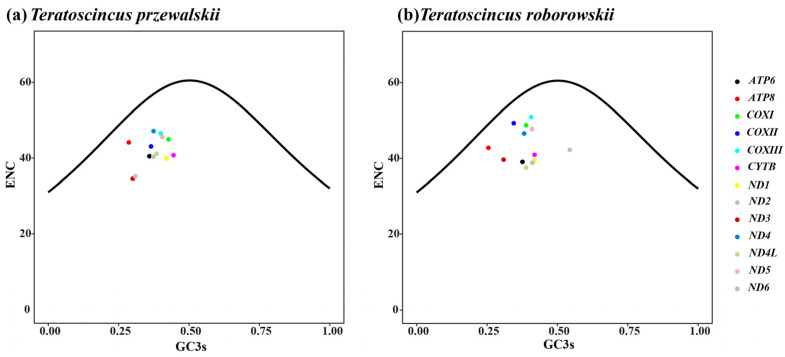
ENC plots for codon preferences in *T. przewalskii* (**a**) and *T. roborowskii* (**b**).

In order to further investigate the influencing factors of the codon usage bias, an analysis was conducted of the correlation between the GC content at the third codon positions of the synonymous codon and the effective number of codons (ENC). The distribution of the data points along the standard curve indicates that the codon bias is yielded by mutation. Otherwise, if the points are observed to be distributed away from the standard curve, this suggests that the codon bias is predominantly shaped by natural selection rather than mutation bias. The results obtained from the analysis of the PCGs of *T. przewalskii* and *T. roborowskii* indicated that their distribution was mostly distributed away from the standard curve. This finding suggests that the formation of codon preference in both wonder gecko species was mainly influenced by natural selection, and not only by mutation bias (see Figure 7).

### 3.6. Phylogenetic Relationships

**Figure 8:**
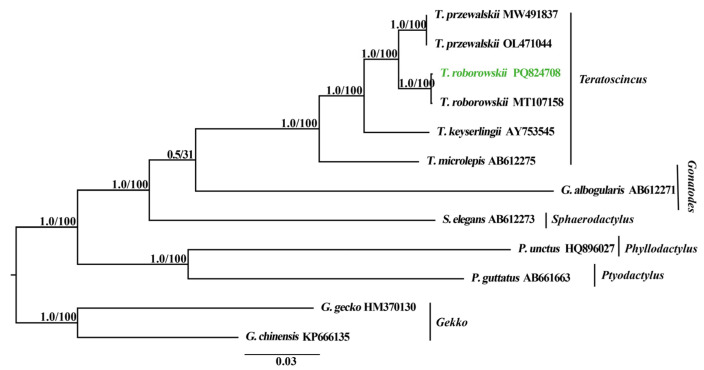
Phylogenetic trees inferred from Bayesian inference (BI) and maximum likelihood (ML) approaches on complete mitochondrial genomes of eight individuals of Sphaerodactylidae and two species of Phyllodactylidae, with two species in Gekkonidae used as outgroups for rooting the tree. Node numbers indicate a posterior probability/UFBoot values. GenBank accession number for the published sequence of each taxon is appended. The taxon highlighted in green represents the individual of *T. roborowskii* that has been sequenced and analyzed in this study.

The results of the BI and ML approaches presented a consistent topological structure (Figure 8). The posterior probability (PP) values of the BI tree and the UFBoot values of the ML tree are shown in Figure 8. Consistent with previous studies [3,4], the phylogenetic tree obtained in this study confirms the monophyly of Sphaerodactylidae, Phyllodactylidae, and Gekkonidae. This result aligns with those of Pyron et al. [41], where Gekkonidae and Phyllodactylidae are sister clades, while Sphaerodactylidae is independent of this sister group. Within Sphaerodactylidae, *Teratoscincus* forms a distinct clade. *T. przewalskii* and *T. roborowskii* are sister species with strong support (PP = 1.0; UFBoot = 100; [1,42]). However, due to lower support value, the sister relationship between *Gonatodes* and *Teratoscincus* remains unresolved (PP < 0.95; UFBoot < 50).

## 4. Discussion

### 4.1. Mitochondrial Genome Organization and Composition

The analysis revealed that the number and order of genes in the mitochondrial genomes of *T. przewalskii* and *T. roborowskii* were consistent with the typical mitochondrial genomes of vertebrates. In accordance with the majority of vertebrates, the mitochondrial genomes of *T. przewalskii* and *T. roborowskii* comprise thirteen PCGs (*ATP6*, *ATP8*, *COI*-*III*, *ND1*-*6*, *ND4L*, and *CYTB*), two rRNAs, twenty-two tRNAs, and two non-coding regions (the control region (D-loop) and origin of replication on the light-strand (OL)). No gene rearrangement was identified, suggesting that genes within the genus *Teratoscincus* may exhibit a high degree of conservation. This specific mitochondrial genome feature has previously been observed in mammals, arthropods, and some reptiles [43,44,45,46,47]. Among the PCGs, the majority were encoded on the heavy strand, with the exception of *ND6*, which was located on the light strand. The existence of varying degrees of genetic overlap between genes of the two species has been demonstrated, enabling a limited number of base loci to carry more genetic information.

The mitochondrial genes of *T. przewalskii* and *T. roborowskii* showed a positive AT-skew and a negative GC-skew, including PCGs, tRNA, rRNA, and the CR. A similar pattern was observed in other species of the genus *Teratoscincus*, which exhibited a slight positive AT-skew and a strong negative GC-skew, indicating the clear bias towards the utilization of A and T in the genus *Teratoscincus*.

RSCU analysis of the two species also exhibited higher RSCU values ending in A/U than those ending in G/C, suggesting a bias in the utilization of A and T in relative synonymous codons. Chen et al. [48] hypothesized that this base usage preference may result from the adaptive evolution of the mitochondrial genome or a compositional preference for high A/T ratios. In addition, it has been shown that AT-bias exists to varying degrees in most reptile families [1,43]. Hassanin et al. [49] hypothesized that this preference for the composition of the A/T nucleotides may be influenced by certain selective pressures, such as mutational pressures and natural selection pressures. Furthermore, incomplete termination codons, including a single T, or an incomplete TA, have also appeared in the two species. Ojala et al. [50] demonstrated that this phenomenon of incomplete termination codons may exert crucial effects on cleavage, transcription, and polyadenylation of multiple cis- and trans-transcripts.

### 4.2. Selection Pressure on PCGs

The purifying selection of PCGs has been recognized as a prevalent phenomenon in most postnatal animals [51]. The present study utilized the *Ka*/*Ks* value as a metric to analyze the pressure on mitochondrial PCGs. The *Ka*/*Ks* value of PCGs was less than 1 and distinct, suggesting that most of the genes were under purifying selection, and the existence of divergent functional constraints among the genes [52]. This finding suggests that the overall evolutionary trend is to retain mutations that do not change the function of the encoded amino acid. The lowest *Ka*/*Ks* value of the *COXI* may be at-tributed to the presence of functional sites associated with species survival adaptations that evolve more slowly [21]. Such as Castoe et al.’s study showed that the *COXI* gene could be intricately linked to the broader context of convergent molecular evolution, particular in snakes and agamid lizards [53]. Conversely, the *Ka*/*Ks* value of the *ND6* of *T. roborowskii* was considerably higher than that observed in *T. przewalskii*, approaching 1. This finding suggests that the rates of non-synonymous and synonymous substitutions of the *ND6* in *T. roborowskii* were converging. It is hypothesized that differences in the selection pressures acting on the PCGs between the two species may contribute to the maintenance of non-synonymous substitutions at optimal rates. This may further facilitate moderate species differentiation and prevent the occurrence of fitness reduction due to excessive substitution [54,55].

Similarly, the pairwise mitochondrial genomes analysis of four *Teratoscincus* species also revealed that *ND6* exhibited elevated rates of evolution, in conjunction with *ATP8*, which also exhibited similarly elevated rates of evolution of (Figure 6). This finding was further confirmed by the analysis of the nucleotide diversity (Pi) value of the PCGs. This suggests a high degree of mutational variation in *ND6* among different species of the genus *Teratoscincus*, indicating that *ND6* is a rapidly evolving gene. In the genus *Teratoscincus*, the ND6 gene likely plays a critical role in survival adaptation. This is supported by studies in toad-headed lizards (*Phrynocephalus*), where the *ND6* gene exhibits signatures of positive selection during evolution, with selected sites mapping to functionally important structural domains. These findings suggest that *ND6* may contribute to environmental adaptation mechanisms in Squamate reptiles [56]. The *ND6* gene in *T. roborowskii* had a slower evolutionary rate compared to its counterpart in *T. przewalskii* (see Figure 5 and Figure 6). This finding suggests that, compared to other PCGs, *ND6* experienced more relaxed selective constraints, which allowing for the accumulation of more mutations. A similar situation of selection pressure was observed in species of Ring-Necked Pheasant (*Phasianus colchicus*) [57], *Dawkinsia filamentosa* and *Pethia nigrofasciata* [58].

### 4.3. Evolutionary Dynamics of Mitochondrial Genes in Teratoscincus

The process of genetic drift and mutation are known to promote the evolution of mitochondrial genes, while purifying selection is responsible for maintaining their function [49]. AT/GC-skew is frequently considered a reliable indicator of the relative abundance of the various bases in mitochondrial DNA and of the evolutionary pressures [59]. As shown in Figure 5, *ND6* and *ATP8* had a greater fluctuation in AT/GC-skew values, suggests that natural selection and mutational pressure on these genes may differ significantly from those observed in other genes. Further exploration of the factors influencing codon usage bias in *T. roborowskii* and *T. przewalskii* indicated that the nucleotide bias situation in both wonder geckos was primarily influenced by natural selection [60,61]. Mitochondrial genomes are implicated in energy metabolism pathways and are subject to multiple environmental pressures in order to meet the metabolic requirements of a species in its environment. It has been demonstrated that certain environmental stresses can promote the adaptive evolution of mitochondrial genes [62]. Consequently, evolutionary selection often acts on environmentally relevant mitochondrial PCGs to enhance the likelihood of mitochondrial genome adaptation to new environments [62,63]. *ND6* is located in the inner mitochondrial mem-brane and functions as the cofactor of NDH. It has been demonstrated that *ND6* is in-volved in the catalysis of NADH dehydrogenase activity and also in the assembly of NADH to ubiquinone and the mitochondrial respiratory chain complex I [51,62].

*T. przewalskii* is a species of desert lizard and is widespread in arid desert dunes in northwestern China. In contrast, *T. roborowskii* is endemic to the Turpan Basin in Xinjiang [1,8], an area characterized by its extremely hot and arid environment [64]. A comparison of *T. przewalskii* and *T. roborowskii* reveals that the former exhibits a higher rate of non-synonymous substitutions in the *ND6*. It is hypothesized that the accelerated evolutionary rate may be attributable to more pronounced selective pressures experienced by *T. roborowskii* within its more distinct environmental conditions. This could drive rapid adaptation to ensure survival and reproduction. The increased rate of non-synonymous substitutions in *ND6* gene may accelerate the fixation of favorable mutations and the elimination of unfavorable ones, allowing the species to adapt to these stronger selective pressures [65]. Consequently, the evolution of the mitochondrial genomes of *T. przewalskii* and *T. roborowskii* displays their differential niche adaptation strategies. *ND6* gene in both *T. przewalskii* and *T. roborowskii* has undergone the various degrees of purifying selection. Consequently, *ND6* gene has the potential to serve as an important genetic marker in further population genetics studies, particularly those focusing on genetic differentiation and local adaptation [66].

### 4.4. Phylogeny of Teratoscincus

Phylogenetic analyses based on the complete mitochondrial genomes statistically recovered the higher-level relationships among *Teratoscincus* (Figure 8). In this study, the outgroup, Gekkonidae, and Phyllodactylidae were identified as sister clades, with Sphaerodactylidae forming a sister group to these families. This finding is in agreement with previous molecular studies [3,41].

Within Sphaerodactylidae, *Teratoscincus* forms a distinct clade, with *T. roborowskii* and *T. przewalskii* identified as sister species. This outcome is not in alignment with the conclusions Yu et al. [12] and Ma et al. [25], but it is in accordance with earlier studies on their phylogenetic systematics [2,23,42]. The observed discrepancy maybe due to the limited number of mitochondrial genomes from the genus *Teratoscincus* employed in constructing the phylogenetic tree, a factor that may have introduced a degree of bias into the results.

## 5. Conclusions

In this work, the mitochondrial genome of *T. roborowskii* was characterized, and comparative analyses were performed within the genus *Teratoscincus*. Our findings reveled that genome size, genome order, intergenic overlap, base composition, and codon usage were conserved among *Teratoscincus* species. However, most PCGs exhibited low evolutionary rates, with *ATP8* and *ND6* being exceptions, displaying faster rates. Of particular note was the finding that the *ND6* gene in *T. roborowskii* had a significantly higher evolutionary rate compared to its counterpart in *T. przewalskii*, suggesting the presence of stronger selection pressures in the former. Phylogenetic analyses supported the independence of Pachypodidae and *Teratoscincus*, and confirmed the sister-taxon relationship between *T. przewalskii* and *T. roborowskii*. These results are in alignment with the majority of previous studies, but the necessity for additional mitochondrial genomes from diverse *Teratoscincus* taxa is highlighted to achieve greater clarify regarding the relationships between the various taxa. These findings promote our understanding of mitochondrial genome structure and selection pressures within the genus *Teratoscincus*, contributing to population genetic studies and enriching the mitochondrial gene pool.

## Figures and Tables

**Table 1:** Characterization of base composition of mitochondrial genomes of *T. przewalskii* and *T. roborowskii*.

Region	Size (bp)		A + T Content (%)		G + C Content (%)		AT-Skew		GC-Skew	
	TP	TR	TP	TR	TP	TR	TP	TR	TP	TR
Whole genome	17,184	16649	55.8	56.2	44.2	43.8	0.103	0.089	0.371	0.356
PCGs	11,367	11340	56.4	56.5	43.6	43.5	0.026	0.012	0.380	0.366
rRNA genes	2493	2503	54.2	54.6	45.7	45.4	0.240	0.224	0.225	0.207
tRNA genes	1531	1531	56.3	56.2	43.7	43.8	0.058	0.06	0.001	0.004
CR	1774	1247	54.1	56.8	45.9	43.2	0.083	0.067	0.315	0.343

## Data Availability

The data supporting the results of this study can be found in the manuscript. The sequences generated during this study have been deposited in GenBank (https://www.ncbi.nlm.nih.gov/genbank/ (accessed on 2 April 2025)) under accession numberPQ824708.

## Supplementary Materials

The following supporting information can be downloaded at: https://www.mdpi.com/article/10.3390/genes16050531/s1. Table S1: Taxon information of Sphaerodactylidae, Phyllodactylidae, and Gekkonidae species analyzed in this paper with GenBank accession numbers. Table S2. Characteristics of mitochondrial genomes of *T. przewalskii* and *T. roborowskii*. Table S3. AT-skew and GC-skew of mitochondrial genomes of *T. przewalskii* and *T. roborowskii*.
