# Supplementary material for: Comparative Mitogenomics of Wonder Geckos (Sphaerodactylidae: Teratoscincus Strauch, 1863): Uncovering Evolutionary Insights into Protein-Coding Genes
Source: Genes (Basel). 2025 Apr 29;16(5):531. doi: 10.3390/genes16050531 (PMC12111026; doi:10.3390/genes16050531)
Supplement: Supplementary file 1 [file genes-16-00531-s001.zip › Supplementary_Table_S1.pdf]

Table S1. Taxon information of Sphaerodactylidae, Phyllodactylidae and Gekkonidae species analyzed in this paper with GenBank accession numbers.

Taxon	Family	Subfamily	Accession Number	Length(bp)	Reference
Teratoscincus keyserlingii	Sphaerodactylidae	*Teratoscincus*	AY753545	18400	[2]
Teratoscincus microlepis	Sphaerodactylidae	*Teratoscincus*	AB612275	16995	/
Teratoscincus przewalskii	Sphaerodactylidae	*Teratoscincus*	MW491837	16779	/
Teratoscincus przewalskii	Sphaerodactylidae	*Teratoscincus*	OL471044	17184	/
Teratoscincus roborowskii	Sphaerodactylidae	*Teratoscincus*	KP115216	16635	/
Teratoscincus roborowskii	Sphaerodactylidae	*Teratoscincus*	LXJ1507	16649	This study
Gonatodes albogularis	Sphaerodactylidae	*Gonatodes*	AB612271	16830	/
Sphaerodactylus elegans	Sphaerodactylidae	*Sphaerodactylus*	AB612273	17500	/
Gekko chinensis	Gekkonidae	*Gekko*	KP666135	17906	[60]
Gekko gecko	Gekkonidae	*Gekko*	HM370130	16591	/
Phyllodactylus unctus	Phyllodactylidae	*Phyllodactylus*	HQ896027	16881	[61]
Ptyodactylus guttatus	Phyllodactylidae	*Ptyodactylus*	AB661663	17662	/
