# Supplementary material for: Comparative Mitogenomics of Wonder Geckos (Sphaerodactylidae: Teratoscincus Strauch, 1863): Uncovering Evolutionary Insights into Protein-Coding Genes
Source: Genes (Basel). 2025 Apr 29;16(5):531. doi: 10.3390/genes16050531 (PMC12111026; doi:10.3390/genes16050531)
Supplement: Supplementary file 1 [file genes-16-00531-s001.zip › Supplementary_Table_S2.pdf]

Table S2. Characteristics of the mitochondrial genomes of *T. przewalskii* and *T. roborowskii*

1) *Teratoscincus przewalskii* OL471044

	Position			Codon				
Gene/Element	From	To	Length(bp)	Start	Stop	Intergenic nucleotide	Anticodon	Strand
tRNA-Phe	1	74	74				GAA	H
12S rRNA	75	1022	948					H
tRNA-Val	1023	1088	66				TAC	H
16S rRNA	1089	2633	1545					H
tRNA-Leu2	2637	2711	75			3	TAA	H
ND1	2712	3688	977	ATC	TA			H
tRNA-Ile	3689	3761	73				GAT	H
tRNA-Gln	3761	3832	72			-1	TTG	L
tRNA-Met	3832	3901	70			-1	CAT	H
ND2	3902	4940	1039	ATA	T			H
tRNA-Trp	4941	5008	68				TCA	H
tRNA-Ala	5009	5077	69				TGC	L
tRNA-Asn	5079	5151	73			1	GTT	L
rep-origin	5152	5178	27					H
tRNA-Cys	5179	5244	66			27	GCA	L
tRNA-Tyr	5245	5314	70				GTA	L
CO Ⅰ	5316	6875	1560	GTG	AGG	1		H
tRNA-Ser2	6867	6937	71			-9	TGA	L
tRNA-Asp	6938	7005	68				GTC	H
CO Ⅱ	7006	7690	685	ATG	T			H
tRNA-Lys	7691	7758	68				TTT	H
ATP8	7759	7923	165	GTG	TAA			H

ATP6	7914	8594	681	ATG	TAA	-10		H
COX3	8594	9377	784	ATG	T	-1		H
tRNA-Gly	9378	9444	67				TCC	H
ND3	9445	9790	346	ATA	T			H
tRNA-Arg	9792	9859	68			1	TCG	H
ND4L	9861	10157	297	ATG	TAA	1		H
ND4	10151	11518	1368	GTG	TAA	-7		H
tRNA-His	11524	11592	69			5	GTG	H
tRNA-Ser1	11593	11659	67				GCT	H
tRNA-Leu1	11667	11738	72			7	TAG	H
ND5	11739	13550	1812	GTG	TAG			H
ND6	13543	14064	522	ATG	TAA	-8		L
tRNA-Glu	14065	14132	68				TTC	L
CYTB	14135	15273	1139	ATG	TA	2		H
tRNA-Thr	15274	15342	69				TGT	H
tRNA-Pro	15343	15410	68				TGG	L
CR	15411	17184	1774					H

2) *Teratoscincus roborowskii* PQ824708

Gene/Element	Position		Length(bp)	Codon		Intergenic nucleotide	Anticodon	Strand
	From	To		Start	Stop			
tRNA-Phe	1	74	74				GAA	H
12S rRNA	75	1,020	946					H
tRNA-Val	1,021	1,086	66				TAC	H
16S rRNA	1,087	2,628	1,542					H
tRNA-Leu1	2,632	2,706	75			3	TAA	H
ND1	2,707	3,683	977	ATC	TA			H
tRNA-Ile	3,684	3,756	73				GAT	H
tRNA-Gln	3,756	3,827	72			-1	TTG	L
tRNA-Met	3,827	3,896	70			-1	CAT	H
ND2	3,897	4,935	1,039	ATA	T			H
tRNA-Trp	4,936	5,003	68				TCA	H
tRNA-Ala	5,004	5,072	69				TGC	L
tRNA-Asn	5,074	5,146	73			1	GTT	L
rep-origin	5,147	5,173	27					H
tRNA-Cys	5,174	5,239	66			27	GCA	L
tRNA-Tyr	5,240	5,309	70				GTA	L
CO Ⅰ	5,311	6,864	1,554	GTG	AGA	1		H
tRNA-Ser1	6,860	6,931	72			-5	GCT	L
tRNA-Asp	6,932	7,000	69				GTC	H
CO Ⅱ	7,001	7,685	685	ATG	T			H
tRNA-Lys	7,686	7,750	65				TTT	H
ATP8	7,751	7,915	165	GTG	TAA			H
ATP6	7,906	8,586	681	ATG	TAA	-10		H

COX3	8,586	9,369	784	ATG	T	-1		H
tRNA-Gly	9,370	9,436	67				TCC	H
ND3	9,437	9,782	346	ATA	T			H
tRNA-Arg	9,784	9,851	68			1	TCG	H
ND4L	9,853	10,149	297	ATG	TAA	1		H
ND4	10,143	11,510	1,368	GTG	TAA	-7		H
tRNA-His	11,515	11,584	70			4	GTG	H
tRNA-Ser2	11,585	11,651	67				TGA	H
tRNA-Leu2	11,659	11,731	73			7	TAG	H
ND5	11,732	13,543	1,812	GTG	TAG			H
ND6	13,536	14,057	522	ATG	TAA	-8		L
tRNA-Glu	14,058	14,125	68				TTC	L
CYTB	14,128	15,266	1,139	ATG	TA	2		H
tRNA-Thr	15,267	15,335	69				TGT	H
tRNA-Pro	15,336	15,402	67				TGG	L
CR	15,403	16,649	1,247					H
