# Supplementary material for: Comparative Mitogenomics of Wonder Geckos (Sphaerodactylidae: Teratoscincus Strauch, 1863): Uncovering Evolutionary Insights into Protein-Coding Genes
Source: Genes (Basel). 2025 Apr 29;16(5):531. doi: 10.3390/genes16050531 (PMC12111026; doi:10.3390/genes16050531)
Supplement: Supplementary file 1 [file genes-16-00531-s001.zip › Supplementary_Table_S3.pdf]

Table S3. AT-skew and GC-skew of the mitochondrial genomes of *T. przewalskii* and *T. roborowskii*

	Regions	Size (bp)	T(U)	C	A	G	AT (%)	GC (%)	AT skew	GC skew
T. przewalskii	1st codon position	3789	19.7	27.9	30	22.3	49.7	50.2	0.207	-0.111
	2nd codon position	3789	40.1	28.8	18.2	12.9	58.3	41.7	-0.377	-0.382
	3rd codon position	3789	22.6	33.5	38.5	5.4	61.1	38.9	0.261	-0.724
T. roborowskii	1st codon position	3780	19.9	27.8	29.9	22.4	49.8	50.2	0.2	-0.107
	2nd codon position	3780	40.4	28.6	18.1	12.9	58.5	41.5	-0.38	-0.38
	3rd codon position	3780	23.4	32.8	37.8	6.1	61.2	38.9	0.235	-0.687
